# Suppression of MAPK Signaling and Reversal of mTOR-Dependent MDR1-Associated Multidrug Resistance by 21α-Methylmelianodiol in Lung Cancer Cells

**DOI:** 10.1371/journal.pone.0127841

**Published:** 2015-06-22

**Authors:** Mark Borris Docdoc Aldonza, Ji-Young Hong, Song Yi Bae, Jayoung Song, Won Kyung Kim, Jedo Oh, Yoonho Shin, Seung Ho Lee, Sang Kook Lee

**Affiliations:** 1 College of Pharmacy, Seoul National University, Seoul, Korea; 2 College of Pharmacy, Yeungnam University, Gyeongbuk, Korea; University of Navarra, SPAIN

## Abstract

Lung cancer is the leading cause of cancer-related deaths worldwide and remains the most prevalent. Interplay between PI3K/AMPK/AKT and MAPK pathways is a crucial effector in lung cancer growth and progression. These signals transduction protein kinases serve as good therapeutic targets for non-small cell lung cancer (NSCLC) which comprises up to 90% of lung cancers. Here, we described whether 21α-Methylmelianodiol (21α-MMD), an active triterpenoid derivative of *Poncirus trifoliate*, can display anticancer properties by regulating these signals and modulate the occurrence of multidrug resistance in NSCLC cells. We found that 21α-MMD inhibited the growth and colony formation of lung cancer cells without affecting the normal lung cell phenotype. 21α-MMD also abrogated the metastatic activity of lung cancer cells through the inhibition of cell migration and invasion, and induced G_0_/G_1_ cell cycle arrest with increased intracellular ROS generation and loss of mitochondrial membrane integrity. 21α-MMD regulated the expressions of PI3K/AKT/AMPK and MAPK signaling which drove us to further evaluate its activity on multidrug resistance (MDR) in lung cancer cells by specifying on P-glycoprotein (P-gp)/MDR1-association. Employing the established paclitaxel-resistant A549 cells (A549-PacR), we further found that 21α-MMD induced a MDR reversal activity through the inhibition of P-gp/MDR1 expressions, function, and transcription with regained paclitaxel sensitivity which might dependently correlate to the regulation of PI3K/mTOR signaling pathway. Taken together, these findings demonstrate, for the first time, the mechanistic evaluation *in vitro* of 21α-MMD displaying growth-inhibiting potential with influence on MDR reversal in human lung cancer cells.

## Introduction

Lung cancer is the most common cancer worldwide for decades, and it accounts for approximately 1.38 million deaths each year for both men and women in the United States alone. The prognosis associated with the disease is very poor delaying the diagnosis until late advanced stages and treatment options are limited, resulting in almost 90% death rate due to treatment failure caused by undetected metastasis progression [1]. Natural products have been used as medical therapeutics for centuries with as many as 70% of all drugs approved for clinical chemotherapy as well as for lung cancer treatment between 1981 and 2002 consisting of either natural products or chemical and synthetic derivatives based on natural products. [2]. However, the mechanism by which most natural products exhibit their therapeutic potential is less well understood. Triterpenoids have been taking an increasing attention lately in lung cancer therapeutics because of their reported chemopreventive and therapeutic potential both *in vitro* and *in vivo* [3,4]. 21α-Methylmelianodiol (21α-MMD) is a natural triterpenoid and an isomer of 21-methylmelianodiols first isolated from the fruits of *Poncirus trifoliata* (Rutaceae), which has long been used in Oriental medicine as a remedy for allergic inflammation. In recent reports, 21α-MMD displayed functional anti-inflammatory activities [5]. However, there has been no report further evaluating its anticancer potential and mechanism of action in lung cancer.

Cancer survival-associated signaling pathways, including phosphoinositide 3-kinase (PI3K)/AKT/mammalian target of rapamycin (mTOR) and mitogen-activated protein kinase (MAPK) and cancer metastasis-associated AMPK pathways play pivotal roles in the regulation of drug-induced functional activities such as DNA damage-induced apoptosis, cell growth inhibition, and anti-metastatic/progression utilities [6,7], with pronounced crucial functional regulatory activity in lung cancer cell proliferation and survival [8]. The exact molecular mechanisms responsible for most of the triterpenoid-induced anticancer activities involving these classical pathways have yet to be elucidated in detail to further incorporate therapeutic strategies for better outcomes.

Another pivotal cause of treatment failure in lung cancer is the occurrence of multidrug resistance (MDR), the principal mechanism by which many cancers become resistant to a broad spectrum of chemotherapeutics. PI3K/AKT and MAPKs signaling have been widely involved in the development of MDR in lung cancer. Stimulation of these pathways renders lung tumor cells resistant to cytotoxic chemotherapeutic drugs such as paclitaxel, to further impact cellular function [9,10]. Sensitivity to different chemotherapeutics varies widely from patient to patient. However, one molecular mechanism can be pointed out to effectively design rationale chemotherapeutic combination treatments, that is by targeting the MDR1 (ABCB1) gene encoded P-glycoprotein (P-gp), responsible for pumping out a variety of xenobiotics and endogenous substances from inside to the extracellular region of the cells [11]. Recent evidences have emphasized the interplay between mTOR signaling and P-gp/MDR1-mediated MDR in hepatocellular carcinomas and colorectal cancer [12,13]. These kind of associations have led to functionally characterize the potential regulatory mechanism of targeting the PI3K/AKT and MAPKs pathway and subsequent impairment of P-gp activity [14,15]. In addition, a number of studies have also suggested the development of drugs based from flavonoids and triterpenoids that can target these signals to subsequent form a category of P-gp inhibitors and enhance the activity of several anticancer drugs, such as paclitaxel and doxorubicin [16–18].

The purpose of this study, therefore, was to mechanistically identify the mode of action of 21α-MMD on human NSCLC cells and further relate its regulatory mechanism on cell growth and survival-related signals such as the PI3K/AKT/AMPK and MAPKs with P-gp/MDR1-associated MDR occurrence in a lung cancer phenotype. Characterization of the mechanisms of action of 21α-MMD may lead to new insights of therapeutic development to combat growth, metastatic activity, as well as the occurrence of MDR in human lung cancers.

## Materials and Methods

### Reagents

Trichloroacetic acid (TCA), (3-(4,5-dimethylthiazol-2-yl)-2,5-diphenyltetrazolium bromide (MTT), sulforhodamine B (SRB), propidium iodide (PI), RNase A, paclitaxel, 5-fluorouracil (5-FU), mouse monoclonal anti-β-actin antibody, dichloro-dihydro-fluorescein diacetate (DCFH-DA), rhodamine-123, crystal violet, N-acetyl-L-cysteine (NAC), and other reagents unless otherwise indicated were purchased from Sigma-Aldrich, Inc. (St. Louis, MO, USA). RPMI 1640 medium, fetal bovine serum (FBS), antibiotic-antimycotic solution, and trypsin-EDTA were purchased from Invitrogen (Grand Island, NY, USA). Mouse monoclonal anti-phospho-ERK (Tyr 204), anti-c-myc, and rabbit polyclonal anti-cyclin A, anti-cyclin B1, anti-cyclin E, anti-CDK2, anti-CDK4, anti-Rb and anti-phospho-Rb, anti-ERK 1/2, anti-p38 MAPK, anti-phospho Akt, anti-Akt, anti-phospho-mTOR, anti-mTOR were purchased from Santa Cruz Biotechnology (Santa Cruz, CA, USA). Mouse monoclonal anti-phospho-JNK/SAPK (Thr 183/Tyr 185), anti-JNK/SAPK, anti-phospho-p38 (Thr 180/Tyr 182), anti-AMPK, anti-phospho-AMPKα (Thr 172), anti-PI3K, and anti-phospho-PI3K p85 (Tyr 458) were purchased from Cell Signaling Technology (Danvers, MA, USA). Alexa Fluor 488-labeled chicken anti-rabbit IgG was purchased from Invitrogen (Carlsbad, CA, USA). Mouse monoclonal anti-P-gp and fluorescein isothiocyanate (FITC)-labeled monoclonal anti-P-gp was purchased from Abcam (Cambridge, MA, USA). The cationic lipophilic dye tetramethylrhodamine ethyl ester (TMRE) was purchased from Molecular Probes (OR, USA). ON-TARGETplusTM (SMARTpool) mTOR and scramble siRNAs were purchased from Thermo Scientific-Dharmacon RNAi Technologies (Suwanee, GA, USA). The test compound, 21α–MMD, was isolated from the fruits of *Poncirus trifoliata* as previously described [19] and provided by Dr. S.H. Lee, a co-author, at the College of Pharmacy, Yeungnam University, Korea.

### Cell Lines and Culture Conditions

Human lung cancer cells (A549, H460, H1299 H358, and H292), human normal lung cells (L132 and MRC-5), human breast cancer cells (MDA-MB-231), human ductal breast epithelial tumor cells (T47D), human liver cancer cells (SK-HEP-1), and human gastric cancer cells (SNU-638) were provided by the Korean Cell Line Bank (Seoul, Korea). Paclitaxel-resistant A549 cells (A549-PacR) were developed by our group from parental A549 cells through continuous exposure to gradually increasing concentrations of paclitaxel maintaining continuous growth and fine parental-like morphology [20]. The cells were cultured in DMEM (for MRC-5, MDA-MB-231, and SK-HEP-1 cells) and RPMI-1640 (for A549, A549-PacR, H358, H1299, H460, H292, T47D, and SNU-638 cells) supplemented with 10% heat-inactivated FBS and antibiotics-antimycotics (PSF; 100 units/ml penicillin G sodium, 100 μg/mL streptomycin, and 250 ng/mL amphotericin B). The cells were incubated at 37°C and 5% CO_2_ in a humidified atmosphere.

### Cell Proliferation Assays

The cell proliferation was evaluated using MTT colorimetric assay except for the preliminary screening of the anti-proliferative activity of *P*. *trifoliata* active compounds which was carried out by SRB assay. Cells were seeded at a density of 3 to 5 x 10^4^ cells per well in 96-well plates with a total volume of 200 μL/well and were allowed to reattach overnight. After 24 h incubation cells were treated with various concentrations of 21α-MMD, H_2_O_2_, drugs, as individual or in combination, or DMSO control continuously at the indicated time courses. After treatment, the cells were fixed with 10% TCA solution and were subjected for SRB or MTT assays. For MTT assay, cells were incubated in MTT (0.5 mg/mL) containing culture medium at 37°C for 4 h. Supernatant medium was removed and DMSO (200 μL) was added to each well and mixed completely to dissolve MTT. For SRB assay, cells were incubated with 0.4% SRB solution for 30 min. The unbound SRB was removed quickly by washing the wells five times with 1% acetic acid and then air dried. 100 μL of Tris buffer (0.01 M, pH 10.4) was added and shaken gently for 5 min on a mechanical shaker. Absorbance was measured at 570 nm for MTT assay while 515 nm for SRB assay with common background subtraction at 650 nm (zero day or replicate control) by Versamax microplate reader (Molecular Devices Inc., Toronto, Canada). Absorbance values were expressed as a percentage of that for untreated or DMSO control cells, and the concentration of tested drugs which was calculated by the formula % viability = ((Ave. Abs.Drug—Ave.Abs.Zero Day) / (Ave. Abs.Control—Ave. Abs.Zero Day)) x 100. The IC_50_ values were calculated as the drug that inhibits cell proliferation by 50% compared with controls using non-linear regression analysis (percent survival vs. concentration). All experiments were performed using four replicates and repeated at least three times in a parallel manner in every drug/compound concentration.

### Colony Formation Assay

Monolayers of cells were treated for 20 h with 21α-MMD at various concentrations, and then harvested, counted, and seeded to 24-well plates at a density of 1,500 cells per well. After one to one and a half weeks, adherent macroscopic colonies were washed with PBS, fixed using 2% paraformaldehyde and stained with crystal violet (0.5% w/v) and then counted visually or by using Image J software.

### Drug Combination Analysis

Cells were seeded at a density of 5 x 10^4^ cells per well in 96-well plates with increasing concentrations of 21α–MMD, paclitaxel, and 5-FU either alone or in combination at their equipotent molar ratio concomitantly. Growth inhibitory activity of the combinations was measured by MTT assay. The combined effects were analyzed using the median effect analysis method and by the calculation of the combination index (CI) using the equation: CI = D_1_/(D_x_)_1_ + D_2_/(D_x_)_2_, where D_1_ and D_2_ are the concentrations of combined compound and drug that achieved the expected effect, and (D_x_)_1_ and (D_x_)_2_ are the concentrations that achieve similar effects when the compounds are used alone. 50% inhibition was chosen as the indicator of efficacy. The calculated CI was then compared with reported reference values [21].

### Cell Cycle Analysis and BrdU Incorporation Assay

Analysis on the effects of 21α–MMD on cell cycle distribution was performed by the method previously described [22]. Cells were treated with or without 21α–MMD for 24 h in 10% FBS-supplemented medium. The cells were harvested, washed twice, and fixed in 70% cold ethanol overnight at -20ºC. Ethanol-fixed cells were pelleted, washed with ice-cold PBS, and resuspended in staining solution containing 50 μg/mL PI, 0.1% Triton-X-100, 0.1% sodium citrate, and 100 μg/mL RNase. After 1 h of incubation at room temperature in the dark, the fluorescence-activated cells were sorted and cellular DNA content analyzed by flow cytometry using the FACSCalibur flow cytometer (BD Biosciences, San Jose, CA, USA) equipped with an argon laser and data were evaluated using CellQuest 3.0.1 software (Becton-Dickinson, Franklin Lakes, NJ). At least 20,000 cells were used for each analysis. Changes in the percentage of cell distribution at each phase of the cell cycle were determined and results are displayed as histograms. The proportion of cells in different phases of the cell cycle was then recorded with at least triplicate and expressed as mean ± SD. Additionally, a colometric BrdU incorporation assay (Abcam) was conducted to measure the rate of DNA synthesis. Briefly, cells were treated with or without 21α–MMD for 24 h in the same medium as above. BrdU is added to cells cultured in microplates followed by 4 h incubation to incorporate BrdU into the DNA of proliferating cells. Culture supernatant was removed followed by fixation. Cells were then incubated with an anti-BrdU antibody conjugated to peroxidase. Bound BrdU is detected by a substrate reaction and quantified by absorbance measurement at 350 nm.

### Measurement of Intracellular ROS

Intracellular ROS was measured by flow cytometry (Becton Dickinson, FACSCalibur) as described previously [23]. Briefly, cells were seeded at a density of 1 x 10^4^ cells/mL in sterile 60 mm dish for 24 h and were treated with or without 21α-MMD for 24 h to detect ROS changes. Cells were harvested and washed twice and stained with 1 mL of DCFH-DA at 10 μM concentration, which was used as the probe to assess the ROS production. Cells were then incubated at 37ºC for 40 min and the peak excitation wavelength for oxidized DCFH-DA at 488 nm with emission at 525 nm were analyzed by flow cytometry. ROS production was expressed as mean fluorescence intensity (MFI) calculated through Cell Quest software. Detection of ROS was also examined by fluorescence microscopy. Briefly, cells were seeded at the density of 5 x 10^4^ cells/mL in cover slips of dishes and incubated overnight for attachment. Cells were then treated with various concentrations of 21α–MMD or NAC, individually or in combination, for 24 h at 37ºC. Cover slips were removed from dishes and cells were stained with 40 μM DCFH-DA for 40 min. The cells were washed with PBS twice to remove excess dye. Cover slips were mounted on glass slides and images were taken using a fluorescence microscope at 200X magnification.

### Measurement of Mitochondrial Transmembrane Potential (∆ѱm)

Changes in the mitochondrial potential were assessed using the fluorescent potentiometric dye TMRE. The cationic lipophilic dye TMRE works by entering the cell in the form of an ester which is subsequently hydrolyzed and converted to tetramethylrhodamine, which is reversibly accumulated in the negatively charged mitochondrial matrix depending on mitochondrial transmembrane potential exhibiting potential-dependent accumulation in mitochondria. To analyze the ∆ѱm after 21α-MMD treatment, cells were growth at a density of 1 x 10^5^ cells per well for 24 h in 24-well sterile culture plate treated with or without 25 μM of 21α-MMD for 24 h. Twenty minutes prior to the end of incubation period, cells were washed with PBS and TMRE dye at 100 nM was loaded for 10 min at 37ºC. Cells were trypsinized and harvested with cold PBS followed by the measurement of fluorescence intensity by flow cytometry (Becton Dickinson, FACSCalibur) and analyzed using CellQuest 3.0.1 software.

### Cell Migration and Invasion Assays

Changes in cell migration were determined and transwell assays without the incorporation of matrigel. For migration transwell assay, cells were seeded onto the upper chambers of 24-transwell plates with 200 μL medium serum starved (without FBS). After treatment with various agents, the cells were left overtime to migrate to the lower chambers containing 800 μL medium with 10% FBS to induce chemotaxis. Migrated cells were visualized by staining with crystal violet (0.5% w/v) after washing with PBS and fixation with methanol. Images were taken and analyzed using JuLI FL microscope aided with Image J software. Cell invasion assay was performed in 24-well transwell plates with polycarbonate (PVDF) filters (8 μm pore size, Corning, USA). Matrigel was diluted to 1 mg/ml with serum-free culture medium and applied on the insert in the upper chambers of the multiwell for invasion assay plate. Cells at the density of 2 x 10^4^ cells per well were seeded into the upper chamber of the transwell unit in 200 μL serum-free culture medium. The lower chamber of the unit was added with 800 μL culture medium supplemented with 10% FBS for chemotaxis induction. After incubation with or without 21α-MMD (5 μM; <IC_50_) for 24 h at 37ºC, the medium in the upper chamber was sucked out, and a cotton swab was used to remove the cells on the upper side of the membrane. Cells that invaded to the underside of the membrane were stained with crystal violet (0.5% w/v) and visualized and scored under JuLI FL microscope. All results are expressed as the percent of migrated or invaded cells as compared with the control (untreated cells).

### Rhodamine-123 (Rho-123) Accumulation Assay

Changes in the efflux function of P-gp in A549-PacR cells were determined parallel to the changes in the accumulation of Rho-123 dye. Cells were seeded into 30 mm dishes at a density of 1 x 10^5^ cells per dish. The cells were pretreated with various concentrations of 21α-MMD for 24 and 48 h. 0.5% DMSO was used as control. After pretreatment, the cells were incubated with 1 μg/mL of Rho-123 dye in culture medium at 37ºC and 5% CO_2_ in the dark for 90 min. After Rho-123 accumulation, the cells were trypsinized from the subconfluent monolayer of cells in culture dishes, and the cell pellet was washed twice with ice-cold PBS. The cells were then analyzed immediately using FACSCalibur (BD Biosciences, San Jose, CA, USA) equipped with a 488 nm argon laser. The concentration of Rho-123 in each sample was determined from the fluorescence measurements by the construction of Rho-123 standard curve. The concentration of intracellular Rho-123 was normalized by the amount of protein and expressed as nmol/g protein. The green fluorescence of Rho-123 was measured using a 530 nm band-pass filter [24]. The efflux function of P-gp was monitored in terms of the decrease of export of Rho-123 with the incorporation of parental A549 cells used as negative control.

### Immunocytochemistry

P-gp localization, immunofluorescence, and DNA fluorescent observation in A549/A549-PacR cells were observed using Zeiss LSM 780 ApoTome microscope (Carl Zeiss, Jena, Germany). The A549/A549-PacR cells were plated into 30 mm dishes with slides and incubated overnight. The cells were pre-treated with various concentrations of 21α–MMD or drug free medium for 48 h. All washes were done with PBS and all incubations were completed at room temperature unless otherwise specified. The cells were washed two times. After treatment incubation, the cells were fixed with 4% paraformaldehyde (in PBS) for 15 min and then blocked with 1% BSA (in PBS) for 1 h at room temperature, the cells were incubated with P-glycoprotein primary antibody at 4°C overnight, then washed for additional two times. Permeabilization with Triton X-100 was not included since the target protein, P-gp, is a transmembrane protein. Following the overnight incubation, cells were incubated with FITC-conjugated secondary antibody for 2 h at room temperature. DAPI (0.5 μg/ml) was used to counterstain the nuclei and stored at 4°C before microscopy.

### Protein Lysates, Western Blotting, and Immunoprecipitation

Cells were seeded in sterile 100 mm dishes for 24 or 48 h and were exposed to various concentrations of 21α–MMD. To determine the levels of target protein expressions, whole-cell extracts were prepared. Treated or non-treated cells were lysed and the protein quantifications were determined using the Bradford assay [25]. Proteins were isolated by lysis buffer (Beyotime, China) [20 mmol/L Tris (pH 7.4), 250 nmol/L NaCl, 2 mmol/L EDTA (pH 8.0), 0.1% Triton X-100, 0.01 μg/mL aprotinin, 0.005 μg/mL leupeptin, 0.4 mol/L phenylmethylsulfonyl fluoride, and 4 mmol/L NaVO_4_] followed by spinning of lysates at 14,000 rpm for 10 min using Thermo Sorvall Legend Micro 21R Refrigerated Centrifuge (Thermo Scientific, Waltham, MA, USA) to remove insoluble material and measured using the Nanodrop 1000 Spectrophotometer (Thermo, USA). The cells were disrupted by sonication and extracted at 4ºC for 30 min. The homogenates were centrifuged at 14,000 rpm for 30 min to remove mitochondria and other insoluble fragments and supernatants were then again centrifuged as above to ensure complete removal of mitochondria. Supernatants from whole-cell lysates were collected and kept at -80ºC. The total protein (100 μg) in each cell lysate was resolved in various percentages of gels matched with the molecular weights of protein targets by sodium dodecyl sulfate-polyacrylamide gel electrophoresis (SDS-PAGE) and was electro-transferred onto PVDF nitrocellulose membranes. The membranes were incubated with blocking buffer (5% non-fat dry milk in phosphate-buffered saline-0.1% Tween 20, PBST, pH 8.0) for 2 h at room temperature and were further incubated with specific antibodies diluted in TBST overnight at 4ºC. After washing with TBST three times, the membranes were incubated with horseradish peroxidase (HRP)-conjugated secondary antibodies for 1 h at room temperature. Visualization of the immunocomplexes was performed using the PowerOpti-ECL-kit (Animal Genetics Inc., Korea) and a LAS 4000 Imager (Fuji Film Corp., Tokyo, Japan).

### RNA Extraction and Real-Time Reverse Transcription-PCR

RT-PCR and real-time RT-PCR were performed to evaluate MDR1 mRNA, cyclin E, and CDK2 mRNA gene expressions after treatment with or without 21α-MMD or siRNA as indicated. Cells at a density of 1 x 10^5^ cells/dish in 100 mm dishes were treated with 21α-MMD for various time courses. After incubation, the cells were washed twice with PBS, and then lysed with TRI reagent for initial RNA isolated step. The RNA was extracted by the addition of chloroform, and the isolated RNA was precipitated with isopropyl alcohol. The RNA pellets were washed with 70% ethanol, air-dried, and then dissolved in nuclease-free water. The absorbance was measured at 260 and 280 nm to determine the concentration and purity of the RNA. The total RNA (1 to 2 μg) was reverse transcribed using AMV reverse transcriptase and oligo(dT)15 primer. A polymerase chain reaction (PCR) was performed in a reaction mixture containing cDNA, 0.2 mM dNTP mixture, 10 pmol of target-specific primers with sequences listed as follows: MDR1 (forward: 5´-CCCATCATTGCAATAGCAGG-3´; reverse: 5´-GTTCAAACTTCTGCTCCTGA-3´), mTOR (forward: 5´-ACTCGCTTCTATGACCAACTGA-3´; reverse: 5´-TTGAAGGTCTCAAACATGAT-3´), cyclin E (forward: 5´-CGGGTCCACAGGATGCGAAGGA-3´; reverse: 5´-CAGGTGTGGGGATCAGGGAGCA-3´), CDK2 (forward: 5´-CATTCCTCTTCCCCTCATCA-3´; reverse: 5´-CAGGGACTCCAAAAGCTCTG-3´), and 0.25 U of Taq DNA polymerase using GeneAmp PCR system 2400 (Applied Biosystems, Foster, CA, USA) to amplify the cDNA. The threshold cycle (Ct) values were determined. The relative mRNA expression levels were normalized to either β-actin (forward: 5´-AGGCACCAGGGCGTGAT-3´; reverse: 5´-GCCCACATAGGAATCCTTCTGAC-3´) or GAPDH (forward: 5´-ATCCCATCACCATCTTCCAG-3´; reverse: 5´-CCATCACGCCACAGTTTCC-3 ´) as indicated, by dividing it by the mean Ct values of the internal control for that sample (∆-Ct). The normalized transcript levels were expressed relative to the sample obtained from the DMSO control (∆∆ - Ct), and the relative RNA expression level was presented as 2-∆-Ct [26]. The PCR products were separated by 2% agarose gel electrophoresis. The gel was stained with 10,000-fold-diluted SYBR safe staining solution and visualized under a UV transilluminator (Alpha ImagerYM, Alpha Innotech Corp., USA). Real-time PCR was conducted using a MiniOpticon system (Bio-Rad, Hercules, CA, USA), using 5 μL of reverse transcription product, 10 μL of iQTMsupermix (Bio-Rad, Hercules, CA, USA), 0.5 μL of primers and probes in a total volume of 20 μL. Standard thermal cycler conditions were employed as follows: 95°C for 5 min before the first cycle, 95°C for 10 sec, 56°C for 30 sec, repeated 40 times.

### Transient Transfection of Small Interfering RNA (siRNA)

The silencing of mTOR with siRNA (25 nM) was achieved by using DharmaFECT transfection reagents following the instructional manual for transient transfection in A549-PacR cells. Scramble siRNA (25 nM), used as control, was also provided. The mixture reagent was added to each well per dish containing 200 μL of serum-free and antibiotic-free medium for a total volume of 300 μL, and the cells were incubated for 4 h at 37°C. An equal volume of medium was then added to each well. Following transfection for 24 h after plating, the cells were enriched with 10% FBS, incubated for another 24 h with 25 μM of 21α-MMD and cells were collected and subjected to analysis. The knockdown of mTOR expression was examined by immunoblotting as described above using anti-mTOR antibody. The mTOR siRNA sequence was 5´-AAGAAUCAAAGAGCAGAGUGC-3´.

### Statistical Analyses

All experiments were repeated at least three times. Data were expressed as the mean ± SD for the indicated number of independently performed experiments and analyzed using a Student’s t-test by non-parametric Mann-Whitney U-test for values that were not normally distributed, one-way ANOVA, and Spearman rank correlation tests where appropriate by GraphPad Prism v5.01 software (GraphPad Software, La Jolla, CA, USA). Values with *p*<0.05 were considered statistically significant.

## Results

### Anti-proliferative effects of compounds from *P*. *trifoliata* on human cancer cell lines

**Fig 1:**
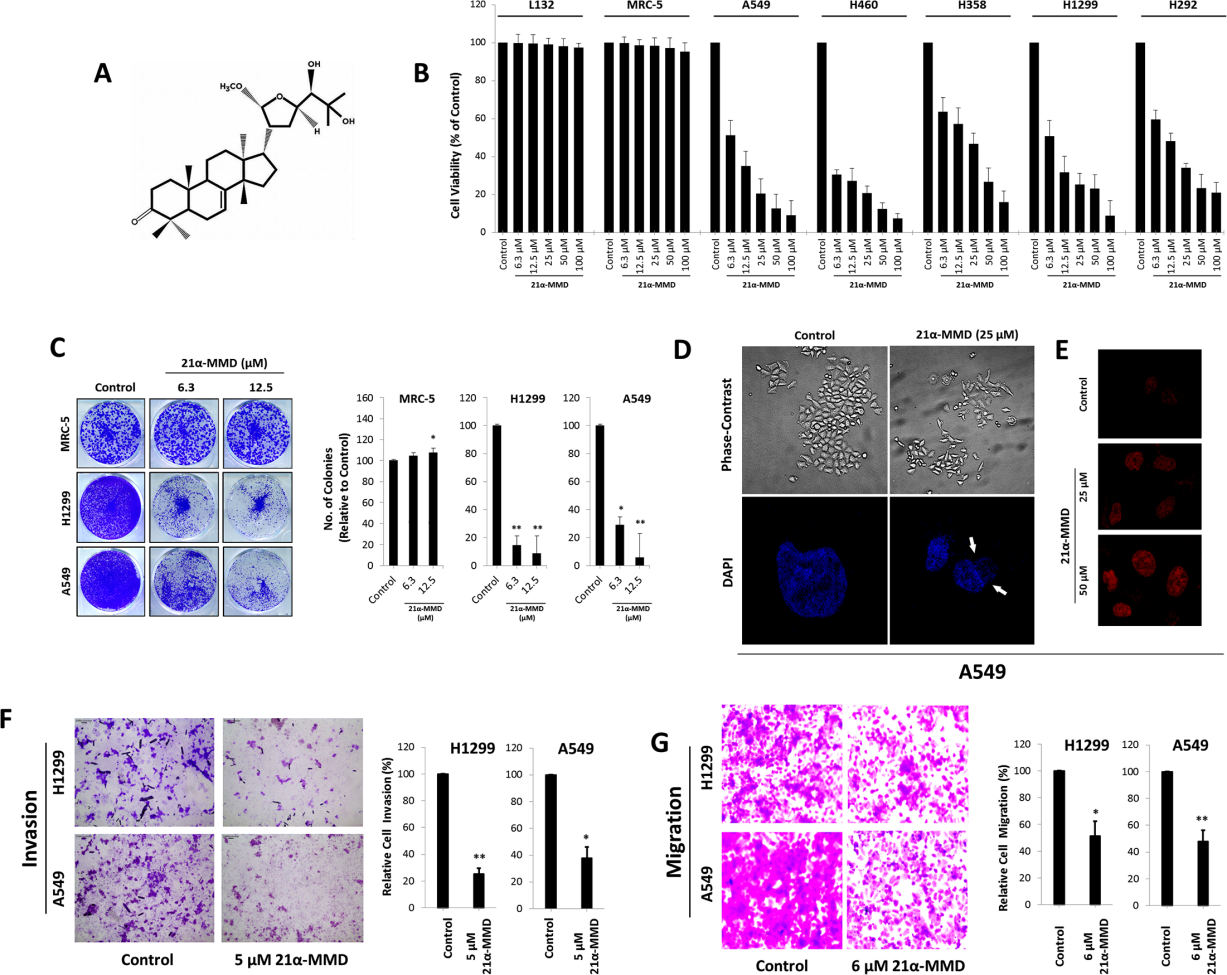
21α-MMD and its mechanistic potential against lung cancer cell growth, migration, and invasion *in vitro*. (A) The chemical structure of 21α-Methylmelianodiol (21α-MMD), a natural triterpenoid isolated from the fruit of *Poncirus trifoliata*. (B) MRC-5 and L132 human normal lung cell lines and A549, H460, H358, H1299, and H292 human lung cancer cell lines were plated on 96-well plate and were treated with varying concentrations of 21α-MMD for 24 h and cell growth was analyzed by MTT assay and plotted as percentage of viable cells. Values are compared to the corresponding control value. (C) Clonal formation growth of indicated lung cancer cells was conducted after a 7-day growth period after a single administration of various concentrations of 21α-MMD. Images on the left displayed are crystal violet stained colonies while on the right are graphs representing count measurements of the colonies. (D and E) Phase-contrast microscopy was conducted on cells after exposure to 25 µM 21α-MMD to identify changes in the morphology and the DNA was observed by DAPI (lower left) and by PI (right) staining observed by confocal microscope. (F) H1299 and A549 cells were incubated with 5 μM 21α-MMD for 24 h followed by cell invasion analysis. Matrigel was diluted with serum-free culture medium and applied on the insert in the upper chambers of the multiwell and the cells were incubated to invade. Invaded cells were stained with crystal violet for apparent detection with a phase-contrast microscope. (G) Migration of cells was analyzed with the same method as in F but except without matrigel inclusion. H1299 and A549 cells were incubated with 6 μM 21α-MMD for 24 h. Columns indicate mean ± SD. (*p<0.05; **p<0.01)

Coumarins and triterpenoids from the fruits of *P*. *trifoliata* have been previously described to exhibit potent anticancer effects against a variety of cancer cell models [27–29]. Based on this information, the *in vitro* cytotoxic activities of 13 compounds isolated from the fruits of *P*. *trifoliata* against MDA-MB-231, T47D, SNU-638, SK-HEP-1, and A549 human cancer cell lines were evaluated using SRB assay. As shown in Table 1, triterpenoids showed the most promise by inhibiting a panel of cancer cells with IC_50_ values in less than 50 micro-molar ranges. Among the test compounds, triterpenoids 25-methoxyhispidol A, 21α-methylmelianodiol (21α-MMD, Fig 1A), and 21α, 25-dimethylmelianodiol exhibited the most active anti-proliferative activity. The data suggested that the anti-proliferative activity of 21α-MMD is potent when tested on A549 human lung cancer cells, which directed us to further study its mechanism of action in a larger panel of NSCLC cell models.

**Table 1:** Anti-proliferative activity of compounds derived from *Poncirus trifoliate*.

Compound	IC_50_ Values (μM)				
	MDA-MB-231	T47D	SNU-638	SK-HEP-1	A549
Poncirin	>100	>100	>100	>100	>100
Naringin	>100	>100	>100	>100	>100
25-Methoxyhispidol A	13.7	8.4	11.9	19.4	12.8
21α-Methylmelianodiol	43.3	13.6	18.3	39.5	16.1
Auraptene	>100	11.5	83.7	82.6	46.7
Hesperidin	>100	>100	99.8	56.9	>100
Hesperidin Methylchalcone	>100	>100	>100	>100	>100
Neohesperidin Dihydrochalcone	>100	>100	>100	>100	>100
21α,25-Dimethylmelianodiol	30.3	9.7	19.9	25.5	14.9
Imperatorin	>100	79.8	87.6	>100	>100
Phellopterin	>100	>100	>100	>100	69.6

### Inhibition of cell proliferation, colony formation, and induction of morphological changes by 21α-MMD in human lung cancer cells

To further elucidate the effects of 21α-MMD on lung cancer cell growth *in vitro*, we used five human lung cancer cell lines (A549, H460, H1299, H358, and H292 cells) and two human normal lung epithelial cell lines (L132 and MRC-5) subjected for MTT assay to evaluate cell growth. The five cell lines represent various subtypes of lung cancer cells (Fig 1B and S1 Fig). Each of the lung cancer cell models showed growth inhibition after 21α-MMD treatment in a concentration- and time-dependent manner with IC_50_ values of 3.1 to 100 μM from 12 to 72 h. The IC_50_ values (μM) of 21α-MMD in these cells at different time courses were as follows: 7.3 (12 h), 6.3 (24 h), 5.4 (48 h), 2.1 (72 h) in A549 cells; 12.7 (12 h) in H460 cells with uncalculated IC_50_ values in 24 to 72 h time courses since the lowest concentration tested, 3.1 μM affected cells leaving 38.3% to 35.3% viability, respectively; 19.7 (12 h), 19.1 (24 h), 14.0 (48 h), 12.0 (72 h) in H358 cells; 12.2 (12 h), 5.6 (24 h) with uncalculated IC_50_ values in 48 and 72 h time courses leaving 41.3% to 38.0% cell viability at 3.1 μM, respectively; 30.0 (12 h), 11.1 (24 h), 4.7 (48 h), 3.2 (72 h) in H292 cells. To recognize whether 21α-MMD can cause unwanted cytotoxicity on normal lung cells, we further evaluated the effects on L132 and MRC-5 cell growth, which showed that 21α-MMD insignificantly affected the growth even when exposed to increasing concentrations and time exposure periods with observed 99.8% to 88.5% viability after treatment for both cell lines. Variations in the IC_50_ values of 21α-MMD can be observed on A549 cells presented in Table 1 and Fig 1B and S1 Fig, respectively despite the same treatment period, were mainly due to the different assays (SRB and MTT) used in evaluating cell proliferation. We next studied the ability of two lung cancer cell lines and one normal phenotype to form colonies in the presence or absence of 21α-MMD for 12 days. 21α-MMD inhibited colony formation of lung cancer cells at concentrations of 6.3 and 12.5 μM. The number of colonies formed was notably inhibited with over 82% and 77% at 12.5 μM in H1299 and A549 cells, respectively, compared to the untreated controls. 21α-MMD did not cause significant inhibition in MRC-5 colony formation with observed increasing number of colonies after treatment (Fig 1C). Additionally, phase-contrast microscopy demonstrated dose-dependent detachment of non-viable cells from the surface of culture plates. Cell shrinkage and membrane blebbing were also observed. The cells treated with 21α-MMD exhibited more heterogenous morphology for 24 h and the number of cells was decreased in comparison with control cells. A large fraction of treated cells demonstrated detachment and cytoplasmic condensation leading to rounding. Proportion of cells with abnormal morphology decreased in parallel after treatment with 21α–MMD. Condensed nuclei and deformed bodies were also observed using confocal microscopy by staining cells with DAPI or PI (Fig 1D and 1E).

### Suppression of cell migration and invasion by 21α-MMD in lung cancer cells

To examine the effect of 21α-MMD on cell migration and invasion, we conducted chamber matrigel invasion assay in A549 and H1299 cells. 21α-MMD (5 μM) profoundly inhibited the capacity of cell invasion in both cells compared with vehicle-treated control cells, with a 75% less invasive cells in A549 while 72.2% less in H1299 cells for 24 h (Fig 1F). Transwell migration assay was also conducted to determine the effect of 21α-MMD on the cellular migratory activity of H1299 and A549 cells (Fig 1G). 21α-MMD displayed migration inhibitory activity with 51.4% (H1299) and 47.8% (A549) migrated cells relative to their controls set to 100%. These findings suggest that 21α-MMD is able to inhibit the cell migration and invasion in lung cancer cells.

### Induction of G_0_/G_1_ cell cycle arrest and modulation of cell cycle regulatory proteins by 21α-MMD

**Fig 2:**
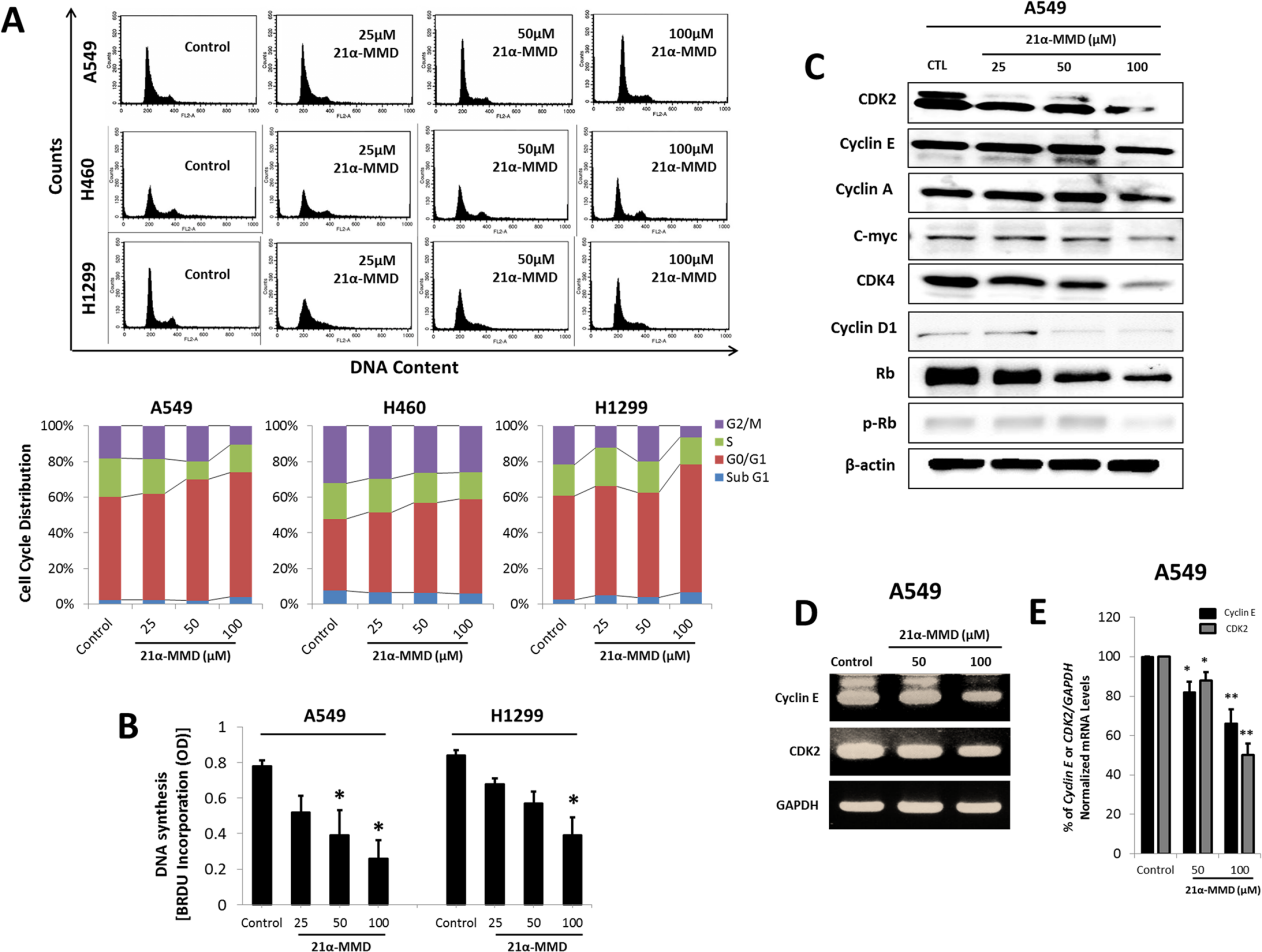
Induction of minimal G_0_/G_1_ cell cycle arrest by 21α-MMD. (A) Flow cytometry analysis was conducted in A549, H460, and H1299 cells after treatment with 21α-MMD for 24 h. (B) A549 and H1299 cells were treated with various concentrations of 21α-MMD for 24 h. DNA synthesis was measured by BrdU incorporation. (C) Cell cycle-related proteins cyclins A, D1, E, CDK2, CDK4, Rb, and phospho-Rb expressions were analyzed by Western blotting after exposure to various 21α-MMD concentrations for 24 h in A549 cells. (D) Cyclin E and CDK2 were selected as 21α-MMD targets to measure mRNA gene expression levels after treatment for 24 h based on the preliminary Western blot analysis results. mRNA levels of cyclin E and CDK2 were measured by RT-PCR and real-time PCR (E). Changes in the mRNA expression of the represented genes were determined by plotting the relative Ct ratio GAPDH, which was used as the internal control in real-time PCR. (*p<0.05; **p<0.01)

To further elucidate the underlying mechanism of 21α-MMD, we investigated whether it can induce cell cycle arrest and alter cell cycle regulatory molecules operative in the G_0_/G_1_ phase transition in various NSCLC cell lines. To determine whether 21α-MMD affects cells in a particular phase of the cell cycle we performed flow cytometry on A549, H460, and H1299 cells after treatment with 25 to 100 μM concentrations of 21α-MMD for 24 h (Fig 2A). In A549 and H460 cells, 21α-MMD caused a rapid but minimal accumulation in the number of cells in the G_0_/G_1_ phase in a concentration-dependent manner, while in H1299 treated cells, there was a slight inconsistent decrease of cells in the G_0_/G_1_ phase at 50 μM by 1.5%, nevertheless, the same phase was arrested with significant accumulation of cells at 100 μM. These findings suggest that 21α–MMD marginally induced G_0_/G_1_ phase cell cycle arrest by potentially and selectively modulating cell cycle regulators in human lung cancer cells. We also incorporated bromodeoxyuridine (BrdU) into A549 and H1299 cells to determine if there would be significant changes in the fraction of S phase cells after treatment with 21α-MMD (Fig 2B). As a result, 21α-MMD caused the inhibition of the S phase of both cells after 24 h treatment. To further examine the association of cell cycle regulatory proteins, Western blot analysis was conducted with the treatment of 21α-MMD (Fig 2C). A significant down-regulation of CDK2, CDK4, cyclin D1, Rb and pRb was found in A549 cells. It was also observed that CDK2, cyclin E, cyclin A, c-myc, and CDK4 were significantly suppressed with 100 μM 21α-MMD treatment for 24 h. Specific suppressive effects of 21α-MMD on cyclin E and CDK2 mRNA gene expressions were also evaluated through RT-PCR. Cyclin E and CDK2 mRNA levels were slightly affected showing detectable changes with 12% to 50% (CDK2), 18% to 34% (cyclin E) fold decrease in A549 cells, respectively. All mRNA levels were normalized to the internal control GAPDH (Fig 2D and 2E).

### Induction of oxidative stress and mitochondrial depolarization through intracellular reactive oxygen species by 21α-MMD

**Fig 3:**
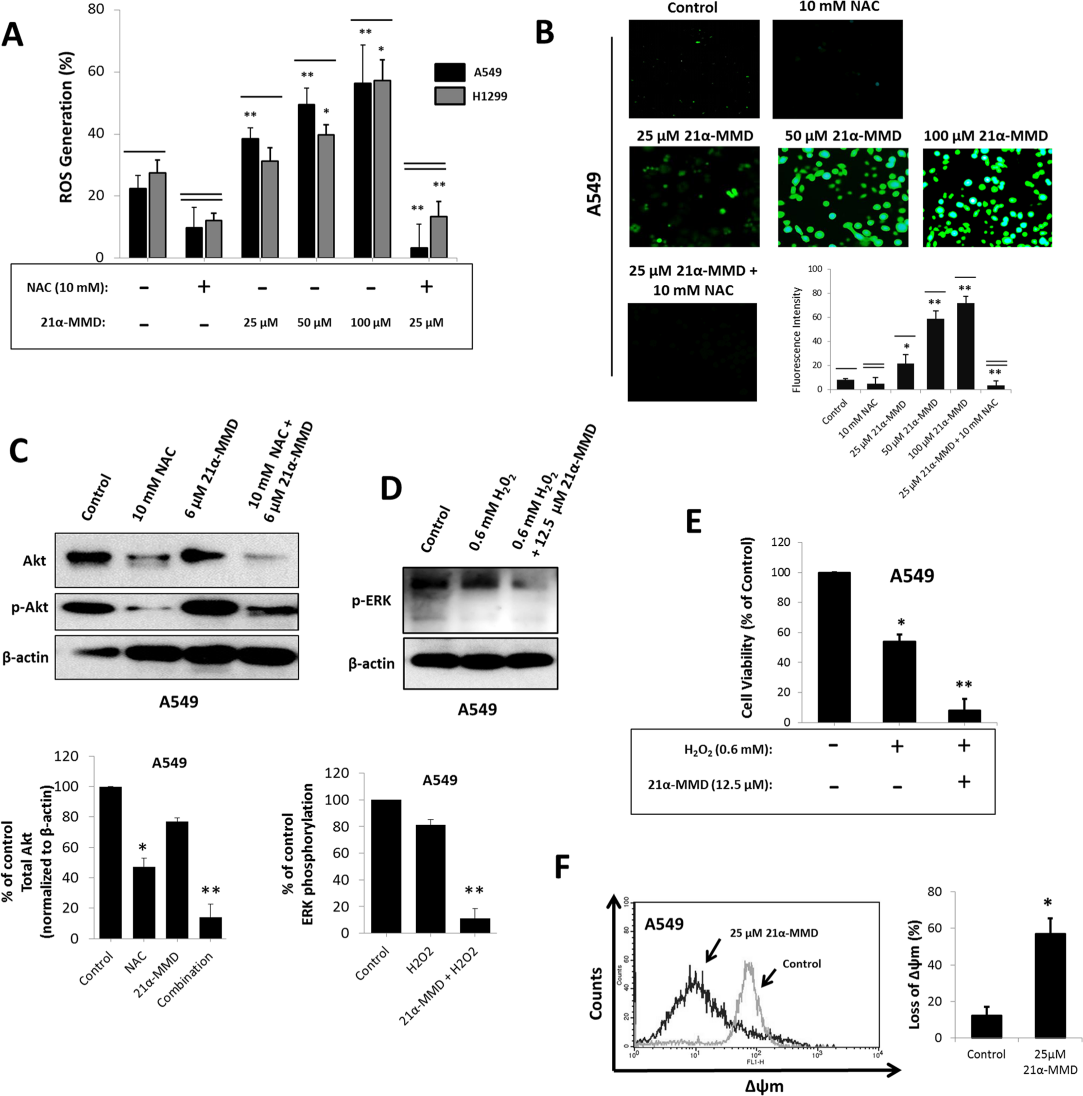
Increased generation of intracellular ROS, loss of mitochondrial transmembrane potential (∆ѱm), and regulation of H_2_O_2_-mediated oxidative stress by 21α-MMD in lung cancer cells. (A and B) After treatment with various concentrations of 21α-MMD for 24 h, the cells were exposed to the oxidative fluorescent dye DCFDA. ROS levels were interpreted as percentage of fluorescent intensity measured by FACS. DCFDA stained cells were observed under fluorescence microscopy to observe intracellular ROS distribution (C) A549 cells were treated with 10 μM NAC and 6 μM 21α-MMD alone or in combination for 24 h. Treated and non-treated cells were subjected to Western blotting for the detection of Akt and phospho-Akt protein expression. (D) A549 cells were treated with 0.6 mM H_**2**_O_**2**_ and 12.5 μM 21α-MMD alone or in combination for 24 h. Treated or non-treated cells were subjected for Western blotting for the detection of ERK and p-ERK protein expression. (E) A549 cells were treated as in (C). Treated or non-treated cells were subjected to MTT assay to examine changes in cell growth rate. (F) A549 cells were treated with 25 μM 21α-MMD for 24 h followed by ∆ѱm determination by flow cytometric analysis. (*p<0.05; **p<0.01)

Regulating changes in the levels of ROS proved to be relatively significant in various cellular functions including cell growth and survival [30]. To further understand the mechanism of the redox regulatory activity of 21α–MMD on A549 and H1299 cells, the levels of ROS after treatment with 21α–MMD (25–100 μM) were determined (Fig 3A). Flow cytometric analysis showed that the proportion of cells with high fluorescence intensity was increased in cells after treatment with 21α–MMD in a concentration-dependent manner for 24 h, indicating that the levels of intracellular ROS in A549 and H1299 were significantly increased with observed 15% to 34% increase in generation levels in A549 cells while 4.5% to 30.8% increase in H1299 cells respective of the increasing concentrations of 21α-MMD from 25 to 100 μM. To further evaluate the involvement of ROS in the activity of 21α-MMD, we incorporated the ROS scavenger NAC with or without 21α-MMD treatment in A549 and H1299 cells for 24 h. Increase of the percentage of ROS production was significantly blocked by NAC at 10 mM concentration with 9.8% and 6.8% generation levels in A549 and H1299 cells, respectively, while 10 mM NAC together with 25 μM 21α-MMD significantly decreased the production with detected 3.1% and 2.4% production in both cells, respectively. Previous studies have revealed that the phosphorylation of Akt, which frequently is hyperactivated in cancer, found to be crucial in ROS-dependent autophagy and contributes to tumor cell resistance to cytotoxic chemotherapies [31]. Akt is also involved in the induction of the accumulation of oxygen radicals, which when exploited can selectively kill cancer cells with high-level Akt [32]. Thus, it is of interest whether 21α-MMD targets Akt phosphorylation to regulate changes in ROS generation. 21α-MMD caused slight inhibition of total Akt expression in A549 cells at 6 μM. However when combined with the ROS inhibitor NAC at 10 mM, total Akt and its phosphorylation were significantly inhibited. This further supports evidences on the involvement of Akt in redox regulation (Fig 3C). Therefore, the activity of 21α-MMD on mitochondrial depolarization might be mediated by ROS and the regulation of its intracellular levels might act as a dependent key factor in affecting the mechanistic activity of 21α-MMD in lung cancer cells. To functionally characterize the ability of 21α-MMD to regulate intracellular ROS generation, we further examined whether 21α-MMD modulates H_2_O_2_-mediated oxidative stress in A549 cells. It has been previously reported that inhibition of ERK significantly increased cell death after H_2_O_2_ treatment in various cell models including epithelial and neuronal cells [33]. We then next identified the possible mechanism for this effect through ROS generation and ERK phosphorylation. 21α-MMD (12.5 μM) significantly down-regulated phospho-ERK expression when combined with H_2_O_2_ (0.6 mM) compared to H_2_O_2_ treatment alone in A549 cells (Fig 3D). Furthermore, it was found that treatment of 0.6 mM H_2_O_2_ with 12.5 μM 21α-MMD increased the oxidative-stress induced cytotoxicity significantly with observed 8% viable cells compared to 54% viable cells when treated 0.6 mM H_2_O_2_ alone, in A549 cells (Fig 3E). These findings suggest that 21α-MMD enhances the H_2_O_2_-mediated cytotoxicity by regulation of ERK phosphorylation. Previous studies showed that differences in the intrinsic mitochondrial membrane potential (∆ѱm) are linked to the sensitivity of tumor cells to various cytotoxic chemopreventive agents [34]. We therefore performed an assay to measure mitochondrial transmembrane potential using the lipophilic cationic dye TMRE to evaluate the effect of 21α-MMD on the mitochondrial membrane integrity of A549 cells. The treatment of 21α-MMD (25 µM) for 24 h was found to induce significant blocking of hyperpolarization of the mitochondrial membrane reflected by decrease in fluorescence intensity compared to untreated cells, suggesting that treatment with 21α-MMD resulted in dissipation of ∆ѱm. After 24 h treatment, the mitochondrial damage resulted in 44.8% reduction in the accumulation of TMRE in the organelle in A549 cells, thus implying a decreased permeability threshold. All mitochondrial integrity values were subtracted and relative to control (Fig 3F). These results suggest that 21α-MMD might act by disrupting the mitochondrial membrane.

### Regulation of PI3K/AMPK/AKT/mTOR and MAPK pathways by 21α-MMD

**Fig 4:**
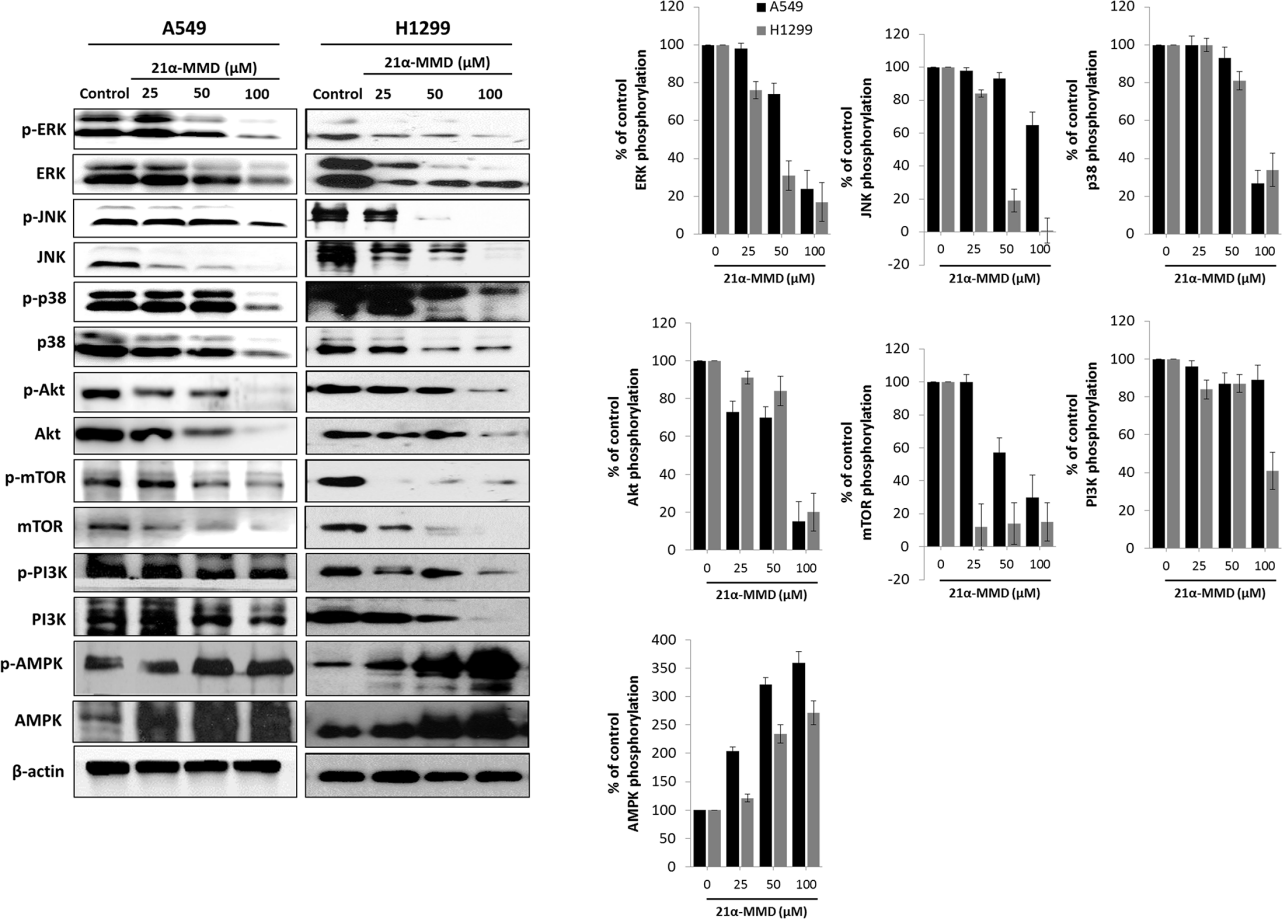
Regulation of the PI3K/AMPK/AKT, mTOR and MAPK signaling pathways by 21α-MMD in lung cancer cells. A549 or H1299 cells were incubated with various concentrations of 21α-MMD for 24 h. Cell lysates were subjected to Western blotting and probed using anti-ERK, anti-JNK, anti-p38, anti-Akt, anti-mTOR, anti-PI3K, anti-AMPK antibodies as well as antibodies for their phosphorylated forms. The β-actin and phospho-protein relevant to the total protein bands confirmed the integrity and equal loading of total and phospho-proteins respectively. All protein levels were normalized to the β-actin levels.

Dysregulation in PI3K/AKT/mTOR, AMPK, and MAPKs pathways are often existent in cancer primarily due to deletion and post-translational modifications [35]. Since the activation of the PI3K/AMPK/AKT/mTOR pathway is shown to cause the development of a more aggressive lung cancer phenotype which correlates to poor prognosis for patients [36], we assessed whether 21α-MMD affects these signals. Strikingly, as for the mTOR signaling, 21α-MMD caused significant concomitant dose-dependent suppressive expressions of PI3K, Akt, mTOR and their respective phosphorylated forms in both A549 and H1299 cells for 24 h. It is known that the inhibition of the mTOR pathway could trigger cancer autophagy [37]. Interplay between the PI3K/mTOR and MAPK pathways has been identified as a critical factor in oncogenesis, specifically to that of lung cancer [38]. Treatment with 21α-MMD resulted in a dose-dependent suppression of total ERK, phospho-ERK, total JNK, phospho-JNK and p38 MAPK expressions in A549 cells with observed significant inhibition of phospho-p38 at 100 μM for 24 h. In H1299 cells, 21α-MMD also significantly down-regulated the activation of protein expressions of ERK, JNK, and p38. Pharmacological activation of AMPK has recently been shown to induce cytotoxicity to many established solid cancer cell lines and human cancer xenografts [39]. We confirmed that 21α-MMD significantly triggered the activation of the AMPK and its phosphorylation in a dose-dependent manner in both A549 and H1299 cells for 24 h (Fig 4). These findings reveal the rationale behind the mechanism of action of 21α-MMD to suppress growth, survival, and metastatic potential of lung cancer cells.

### Potentiation of the cytotoxic activities of paclitaxel and 5-FU in A549 cells and sensitization to anti-cancer drugs in multidrug resistant phenotype by 21α-MMD

**Fig 5:**
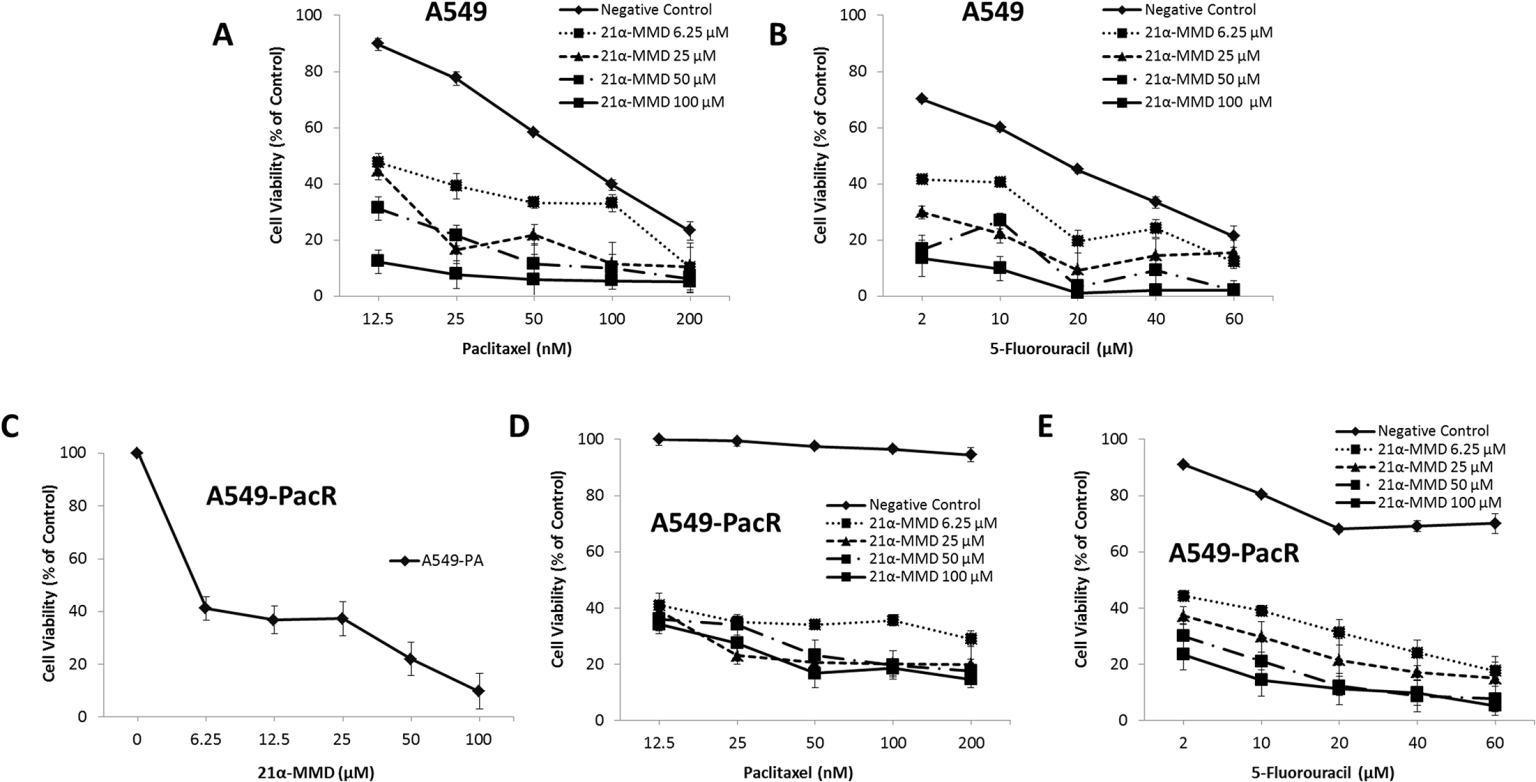
Intensification of paclitaxel and 5-FU cytotoxicity in A549 and A549-PacR cells by 21α-MMD. The cytotoxicity of 21α-MMD (6.25–100 μM), paclitaxel (12.5–200 μM), and 5-FU (2–60 μM) alone or in combination in A549 and A549-PacR cells was determined by MTT assay. Each point indicates the mean ± SD of three independent experiments, performed in triplicate. (A) Effects of 21α-MMD and paclitaxel (B) and 5-FU in A549 cells. (C) Effect of 21α-MMD (D) and paclitaxel (E) and 5-FU in A549-PacR cells. Cells were pretreated with or without 21α-MMD followed by various concentrations of paclitaxel or 5-FU for 24 h. MTT data were presented as the surviving cell viability after treatment regime. A negative control was used which identifies as the respective drug used in combination with 21α-MMD.

We further examined the effect of 21α-MMD in combination paclitaxel and 5-FU, currently used in the clinic for lung cancer treatment, on lung cancer cell growth at 24 h. To search for the optimal concentrations, various concentrations of drugs were tested based on their IC_50_ values. Parental A549 and MDR phenotype A549-PacR cells were used as models to identify whether 21α-MMD can synergize with paclitaxel to either or both produce an effective combination strategy and overcome paclitaxel-resistance. In addition, 5-FU was also employed since it was observed that A549-PacR has a cross-resistance to this drug in the preliminary findings of this experiment. The viability of the cells were examined by MTT assay, which suggested that the concentration groups of 21α-MMD used in this study (6.25 to 100 μM) showed a significant potentiating inhibitory effect to the cell viability of A549 (Fig 5A and 5B) when combined with paclitaxel and 5-FU, respectively. The paclitaxel and 5-FU treatments individually were used as negative controls. The interaction between 21α-MMD and paclitaxel or 5-FU was further evaluated by combination index (CI) analysis. The CI analysis for combining 21α-MMD with cytotoxic agents revealed significant synergy in A549 cells (Table 2). There is a clear synergistic effect in all combinations tested in A549 cells, although with different degree variations from slight to strong synergism with CI values of less than 1, producing a concentration-dependent decrease in the IC_50_ values of paclitaxel and 5-FU in A549 cells. These findings suggest that 21α-MMD might be a potential agent for combination therapy in the clinic. In the context of MDR reversal, 21α-MMD alone significantly inhibited the growth of A549-PacR in a concentration-dependent manner for 24 h (Fig 5C), suggesting a potential MDR modulatory activity. In A549-PacR cells, 21α-MMD significantly sensitized the MDR cells to either paclitaxel or 5-FU with observed dramatic inhibitory shift after treatment in a concentration-dependent manner for 24 h (Fig 5D and 5E). These results demonstrate that 21α-MMD significantly sensitizes P-gp/MDR1-overexpressing A549-PacR cells to anti-cancer agents that are ABCB1 substrates.

**Table 2:** Combinatory effects of 21α-MMD and paclitaxel or 5-FU on A549 cells in a time-course analysis.

21α-MMD (μM)	Paclitaxel (nM)	Time (h)	Combination Index	Description	5-FU (μM)	Time (h)	Combination Index	Description
6.25	12.5	24	0.88	Slight Synergism	2	24	0.82	Moderate Synergism
		48	0.59	Synergism		48	0.41	Synergism
		72	0.89	Slight Synergism		72	0.80	Moderate Synergism
25	25	24	0.52	Synergism	10	24	0.85	Moderate Synergism
		48	0.38	Synergism		48	0.19	Strong Synergism
		72	0.71	Moderate Synergism		72	0.18	Strong Synergism
50	100	24	0.66	Synergism	20	24	0.10	Strong Synergism
		48	0.44	Synergism		48	0.38	Synergism
		72	0.69	Synergism		72	0.22	Strong Synergism
100	200	24	0.61	Synergism	40	24	0.11	Strong Synergism
		48	0.52	Synergism		48	0.21	Strong Synergism
		72	0.87	Slight Synergism		72	0.17	Strong Synergism

### Suppression of expression, function, and transcription of P-gp/MDR1 by 21α-MMD

**Fig 6:**
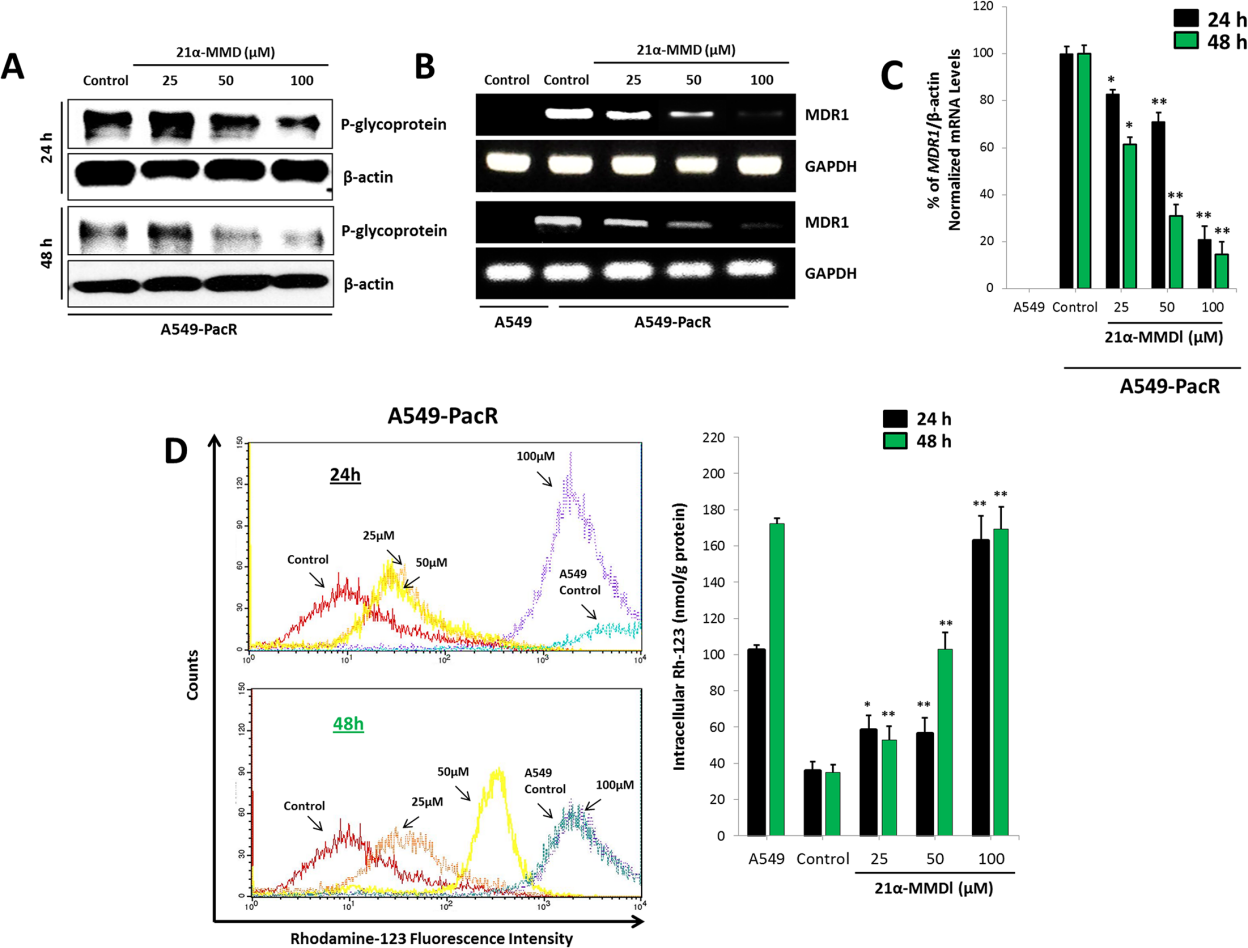
Suppression of expression, function, and transcription of MDR1/P-glycoprotein (P-gp) by 21α-MMD in A549-PacR cells. (A) P-gp protein expression was determined by Western blotting after 24 and 48 h treatment with 21α-MMD at various indicated concentrations (B) The effect of 21α-MMD interaction with MDR1 mRNA gene expression levels in A549-PacR cells was determined by RT-PCR analysis. Cells were treated with 21α-MMD for 24 and 48 h. (C) Effect of 21α-MMD on intracellular Rhodamine-123 (Rho-123) accumulation in A549-PacR was quantitatively measured by flow cytometry. Cells were treated with various concentrations of 21α-MMD for 24 h and 48 h followed by the exposure to 1 μg/ml of Rho-123 dye for 90 min. Parental A549 cells was used as positive control. Each column shows the mean ± SD of three independent experiments, performed in triplicate. (*p<0.05; **p<0.01)

**Fig 7:**
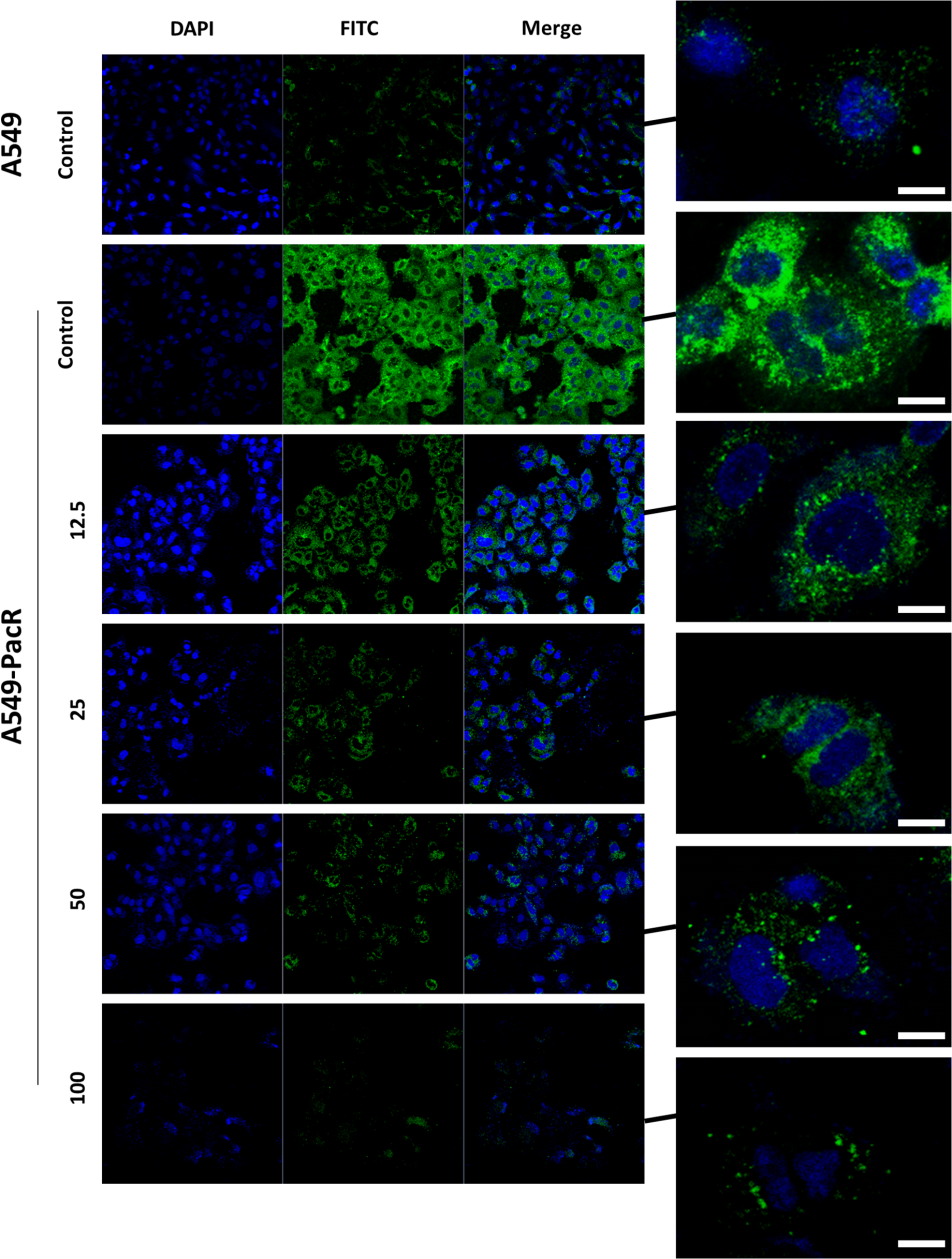
Localization of intracellular P-gp distribution after treatment with 21α-MMD in A549-PacR cells. Confocal analysis of the expression, distribution, and overlapping of P-gp (green) is shown for 12.5–100 μM 21α-MMD treatment in A549-PacR cells. The nuclei were stained with DAPI (blue). Images are representative of three independent experiments. Scale bars, 40 μm.

Overexpression of MDR1 mRNA and P-glycoprotein (P-gp) levels is associated with phenotype multi-drug resistance (MDR). P-gp, encoded by the ABCB1/MDR1 gene, functions as an ATP-driven efflux pump transporter [40,41]. The effect on the expression and efflux pump activity of MDR1/P-gp in A549-PacR cells was examined accordingly after treatment with various concentrations of 21α-MMD. To elucidate potential P-gp suppression activity, P-gp protein expression was examined by Western blotting. P-gp expression was suppressed in A549-PacR in a time-dependent manner at higher 21α-MMD concentrations while revealing less or no expression of P-gp in parental A549 cells (Fig 6A). However, at 25 μM, 21α-MMD caused slight induction of P-gp protein expression which can be attributed to the specific characteristic of the P-gp efflux pump being stimulated by inhibitors at lower cytotoxic doses but significantly inhibited at higher concentrations. One might consider 21α-MMD as in the same category of andrographolide, berberin, glycyrrhizin, etc. by which their characterization as both P-gp (MDR1) inducers and inhibitors are given by their “biphasic protein modulation” [33]. Consistent with this, 21α-MMD significantly suppressed MDR1 mRNA expression and levels in A549-PacR cells in both same manners with observed 17.2% to 69.0% (24 h) and 38.7% to 85.5% fold decrease in mRNA levels (Fig 6B and 6C). To further examine the suppressive effects of 21α–MMD on P-gp expression and whether it is functionally associated with the recovery of drug accumulation in A549-PacR cells, Rho-123 accumulation assay was conducted. Rho-123 dye was used as a substrate to determine the efflux function of P-gp in MDR1/P-gp overexpressing A549-PacR cells since the P-gp-dependent efflux of fluorescent Rho-123 was extensively used in determining efflux from drug-resistant cell lines expressing P-gp. The intracellular accumulation of Rh-123 was detected using flow cytometer. The parental A549 cells, which do not express P-gp, occurred with high accumulation of intracellular Rho-123 (the peaks were shifted to the right side of histogram). In A549-PacR cells, which overexpress P-gp, however, accumulated relatively lower levels of Rho-123 due to the efflux pump action of P-gp. In line with P-gp inhibition, 21α–MMD increased the intracellular accumulation of Rho-123 at higher concentrations in a time-dependent manner indicating that 21α–MMD supports its suppressive effects on P-gp/MDR1 expression and function, revealing elevated P-gp efflux function by 58.24% to 162.8% (24 h) and 52.8% to 169.4% (48 h). Moreover, to examine enhanced Rho-123 efflux, the degree of enhancement by fluorescence intensity was also determined. We found an enhanced uptake of Rho-123 in 21α-MMD-treated A549-PacR cells in a dose-dependent manner for 24 h compared to the non-treated A549-PacR cells with almost no fluorescent visibility while competent when compared to parental A549 cells (Fig 6D). These findings suggest that 21α-MMD inhibits P-gp efflux function in A549-PacR cells. To demonstrate the localization of P-gp in A549-PacR cells, confocal microscopy was also conducted. Immunofluorescence analysis demonstrated that P-gp, which is labeled with green fluorescence (FITC), is mainly overexpressed in the cellular plasma membrane and cytoplasmic regions of A549-PacR cells while less expressed in parental A549 cells (Fig 7). Nuclei of the cells were labeled with blue fluorescence (DAPI). After treatment with 21αα-MMD at various concentrations for 24 h, the amount of P-gp fluorescence was significantly decreased dose-dependently.

### Suppression of MDR1/P-gp function by 21α-MMD through inhibition of mTOR activity

**Fig 8:**
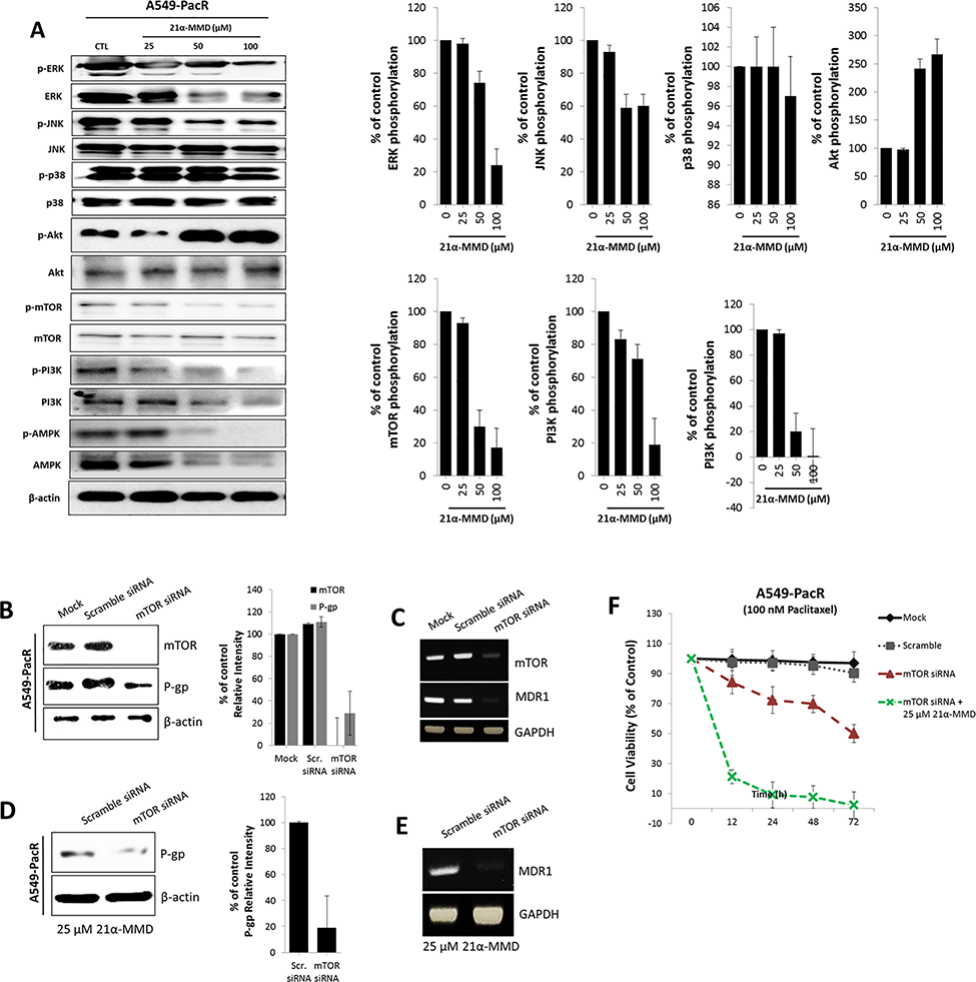
Subsequent downregulation of P-gp/MDR1 expression and inhibitory enhancement of 21α-MMD by stimulating mTOR and related signaling. (A) A549-PacR cells were incubated with 21α-MMD (25–100 μM) for 24 h. Whole-cell lysates were subjected to Western blot analysis using anti-ERK, anti-JNK, anti-p38, anti-Akt, anti-mTOR, anti-PI3K, anti-AMPK and antibodies related to their phosphorylated forms. The β-actin and phospho-protein relevant to the total protein bands confirmed the integrity and equal loading of total protein and phospho-proteins respectively. All protein levels were normalized to the β-actin levels. (B and C) Effect of mTOR knockdown was established first by transiently transfecting mTOR siRNA to A549-PacR cells for 24 h. Scramble siRNA was used as control separated from the mock control. mTOR and MDR1/P-gp protein and mRNA gene expressions were examined by Western blotting and PCR analysis respectively. (D and E) Cells were transiently transfected with mTOR siRNA or Scramble siRNA for 24 h followed by a 24 h exposure to 25 μM 21α-MMD. MDR1/P-gp protein and mRNA gene expression levels were confirmed by Western blotting and PCR analysis respectively. (F) After mTOR siRNA or Scramble siRNA transfections, cells were treated with 100 nM paclitaxel for various time courses and mTOR siRNA was subsequently incorporated with 25 μM 21α-MMD followed by cell viability assessment through MTT assay.

Since activities interplaying with signaling pathways of PI3K/AMPK/AKT/mTOR, and MAPKs can promote cancer chemoresistance and radioresistance [34,35], we assessed whether 21α-MMD affects these pathways in A549-PacR cells. Treatment with 21α-MMD resulted in the suppression of ERK, phospho-ERK and phospho-JNK were suppressed in a dose-dependent manner but with insignificant changes in the expressions of p38, phospho-p38, and JNK (Fig 8A). Pharmacological activation of AMPK has recently been shown to induce cytotoxicity to many established solid cancer cell lines and human cancer xenografts [42,43]. In line with this, 21α-MMD significantly triggered the activation of the AMPK and its phosphorylation in a dose-dependent manner in A549-PacR cells for 24 h. Aside from reports pointing PI3K/Akt/mTOR pathway to be associated with the occurrence of multidrug resistance, it has long been known that inhibition of the mTOR pathway could trigger cancer autophagy [44]. Strikingly, as for the mTOR signaling, 21α-MMD caused significant concomitant dose-dependent suppressive expressions of PI3K, Akt, mTOR and their respective phosphorylated forms in A549-PacR cells for 24 h. Recent evidences show that inhibition of mTOR can overcome cisplatin resistance through subsequent regulation of MDR1 [38,45]. From such perspective, targeting mTOR signaling in multidrug resistant cells with hyper efflux pump activity regulated by P-gp destined for MDR reversal appears consequential. To further elucidate the effect of 21α-MMD on MDR1 promoter activity, A549-PacR cells overexpressing MDR1/P-gp were transfected with mTOR siRNA and were then treated with 25 μM 21α-MMD. The transfection knocked down the expression of mTOR protein and significantly suppressed the P-gp protein and MDR1 mRNA expressions (Fig 8B and 8C). Treatment with 21α-MMD resulted in a dramatic inhibition of MDR1/P-gp promoter activity, indicating that the MDR1/P-gp reversal activity of 21α-MMD could be dependent on mTOR signaling (Fig 8D and 8E). Furthermore, to assess whether mTOR siRNA combined with 21α-MMD treatment caused immobilization of P-gp efflux pump function, we assayed the cell viability of A549-PacR cells after receiving 100 nM paclitaxel treatment for various time courses and evaluated the effects mTOR siRNA with or without 25 μM 21α-MMD. We found significant paclitaxel sensitivity shift of A549-PacR cells from being non-responsive to highly responsive to paclitaxel after receiving mTOR siRNA and the combination with 21α-MMD (Fig 8F). These results revealed that inhibition of PI3K/mTOR pathway is essential for blocking the occurrence of P-gp-associated multidrug resistance in lung cancer.

## Discussion

Herein we report for the first time the mechanistic action of a natural triterpenoid, 21α-MMD on the growth of lung cancer cells. Importantly, 21α-MMD exhibits sub-micromolar efficacy in all lung cancer cell lines tested regardless of their sub-phenotype classification but with greater potential against NSCLC and was highly selective towards them over human normal lung cells. Previous studies reported the anti-inflammatory activity of 21α-MMD by inhibiting nitric oxide production and attenuating LPS-induced inducible nitric oxide synthase (iNOS) and cyclooxygenase (COX)-2 expressions in RAW 264.7 macrophages [46,47]. Apparently, its effect was due to a mechanism involving nuclear factor- κB (NF- κB) activation. Most likely, this was as consequence of its possible anti-oxidative function, which may require cofactors such as tumor necrosis factor-α (TNF-α) and interleukin-1β (IL-1 β) as 21α-MMD was shown to attenuate their mRNA expressions in macrophages [47]. Curious to know whether 21α-MMD can exert anti-oxidative function or in other way support the redox activity against NSCLC cells, we examined its activity on ROS-triggered regulatory utility by which 21α-MMD significantly increased intracellular ROS generation which might be related to Akt signal since an enhanced inhibition was observed after lung cancer cells were treated with relative low-concentration of 21α-MMD and *N*-acetyl cysteine, a radical scavenger. We further identified that 21α-MMD can accentuate the hypoxic effect of H_2_O_2_ on NSCLC cells via the ERK signal. 21α-MMD also lowers mitochondrial membrane permeability, thereby increasing cell sensitivity to oxidative stress which explains the hypoxic accentuating effect of 21α-MMD. Therefore, 21α-MMD generates ROS causing an oxidative stress, regulating the activity of antioxidant defense system and consequent mitochondrial damage evident from the hyperpolarization of the mitochondrial membrane caused by 21α-MMD. In evaluating its mechanistic action on lung cancer cells, we found that 21α-MMD suppressed the growth and clonogenecity, minimally induces G_0_/G_1_ cell cycle arrest, and inhibits the migration and invasion of lung cancer cells. 21α-MMD is also considered to be an attractive chemotherapeutic as a single agent or in combination with paclitaxel or 5-FU against NSCLC cell growth.

A body of evidences suggest that MAPK and AMPK signaling cascades are additional targets affected by the modulatory utilities of oxidative stress [48,49]. MAPK pathways consisting of subfamilies ERK, JNK, and p38, are known to be evolutionarily conserved kinase modules which link extracellular signals to the machinery that controls fundamental cell processes such as growth, migration and apoptosis. Regulation of MAPK pathways and integration between these signals widely vary in different tumors but certainly affects the outcome and sensitivity of cancer cells to drug therapy [50–52]. On the other hand, the activation of AMPK is proved to be essential in progress of various cancer metastases such that of lung cancer, and is mainly involved in differentiation and cell migratory ability of different lung cancer cell phenotypes [53]. Presently, it is still unclear how the signal network between MAPK and AMPK affects regulatory points in coupling the energy status of the cell to the regulation of ROS-triggered metastatic behaviors of cancers and cell survival. Thus, it is of prominent importance to examine drug activities involving these pathways. 21α-MMD exhibited the regulation of MAPK by which the ERK and its phosphorylation were significantly inhibited and the AMPK and its phosphorylation was strikingly upregulated. These data further supports the anti-cancer activity of 21α-MMD in lung cancer cells.

Dysregulation in the PI3K/AKT/mTOR pathway also prove to be critical in MDR modulation in various cancers [54]. Following reports on several anticancer agents inhibiting mTOR signaling and induce autophagy in cancer cells by suppressing major components in the mTOR axis [55]. Due to its mechanistic activity involving these crucial pathways which directly or indirectly interplay with MDR1 activities, we posed a potent functional effect for 21α-MMD to affect such interaction.

The identification and characterization of a compound that reverses MDR1/P-gp has strong implications for both the development of novel chemotherapeutics that can overcome the occurrence of multidrug resistance, a major failure in chemotherapy, and further understanding the interaction networks of P-gp. P-gp expression was extensively demonstrated in several cancers and malignancies and is one factor by which cells acquire multidrug resistance. Among other transport proteins such as multidrug resistance-associated protein-1 (MRP-1) and lung resistance protein (LRP), P-gp is the most extensively examined [56,57]. We found that 2103B0031-MMD exhibits a potential reversal of MDR in paclitaxel resistant lung cancer cells by influencing the abrogation of the expression and function of P-gp/MDR1. 21α-MMD also markedly increased the cytotoxicity of paclitaxel and 5-FU in P-gp overexpressing A549-PacR cells, which indicates that the suppression of MDR1/P-gp function regains the sensitivity of the cells to paclitaxel or 5-FU and illustrates that this activity is a non-specific drug interaction but MDR reversal. In addition, 21α-MMD significantly increases intracellular Rho-123 accumulation in A549-PacR cells, indicating that 21α-MMD suppresses the efflux pump function of P-gp. Furthermore, we found that 21α-MMD down-regulated the expression of P-gp in both mRNA and protein levels and inhibited the MDR1 transcriptional activity and paclitaxel-induced MDR1 promoter activation. These findings additionally confirm that 21α-MMD is capable to suppress MDR1 transcription.

Many evidences demonstrate that PI3K/mTOR signaling is associated with P-gp induction [58–60]. The chemical inhibition of the signaling pathway and mTOR siRNA knockdown exhibits that PI3K/mTOR signaling participates in the induction of P-gp mediated by cholesterol-related molecule [61]. It has also been described previously that treatment with rapamycin, an inhibitor of mTOR, resulted in decreased MDR1 transcription activity in colorectal cancer and eukaryotic cells [62,63]. However, the mechanism on how PI3K/mTOR signaling is activated to directly affect P-gp function and their molecular association is yet to be known. On the other side, modulators of MDR, considering those that inhibit P-gp function, are not effective as expected because, besides ABC transporters, various other mechanisms contribute to drug resistance [64,65]. Approaches to exploit autophagy to overcome MDR have been used as a strategy to potentiate anticancer therapy [66]. Therefore, it was suggested that PI3K/mTOR signaling is a potential target to combat MDR. We demonstrated supporting data that MDR1/P-gp expression was down regulated by mTOR siRNA knockdown and the 21α-MMD treatment caused a significant enhanced mTOR siRNA-mediated down regulating effect through blocking the transcription of P-gp which was confirmed when 21α-MMD sensitized A549-PacR cells to paclitaxel.

In summary, this study demonstrates, for the first time, the anticancer potential of 21α-MMD with a redox regulatory mechanism and also outlines the mechanistic action of 21α-MMD in inhibiting the growth, survival and metastasis of human NSCLC cells and influence a mechanism that results in the influence on suppression of P-gp/MDR1-associated MDR in drug-resistant human NSCLC cells.

## Supporting Information

S1 Fig Suppression of growth of human lung cancer cells by 21α-MMD in dose- and time-dependent manner. L132 and MRC-5 human normal lung cell lines; A549, A549-PacR, H460, H358, H1299, and H292 human lung cancer cell lines were treated with varying micromolar concentrations of 21α-MMD in a time course analysis as indicated. Cell growth was analyzed by MTT assay and plotted as a percentage. The mean values ± SD (n = 3) are shown. Values are compared to the corresponding control value.
(TIF)
