# Correction: Suppression of MAPK Signaling and Reversal of mTOR-Dependent MDR1-Associated Multidrug Resistance by 21α-Methylmelianodiol in Lung Cancer Cells

**DOI:** 10.1371/journal.pone.0133922

**Published:** 2015-07-21

**Authors:** Mark Borris Docdoc Aldonza, Ji-Young Hong, Song Yi Bae, Jayoung Song, Won Kyung Kim, Jedo Oh, Yoonho Shin, Seung Ho Lee, Sang Kook Lee

Fig 8 is incorrect in the published article. Please view the correct Fig 8 here.

**Fig 8:**
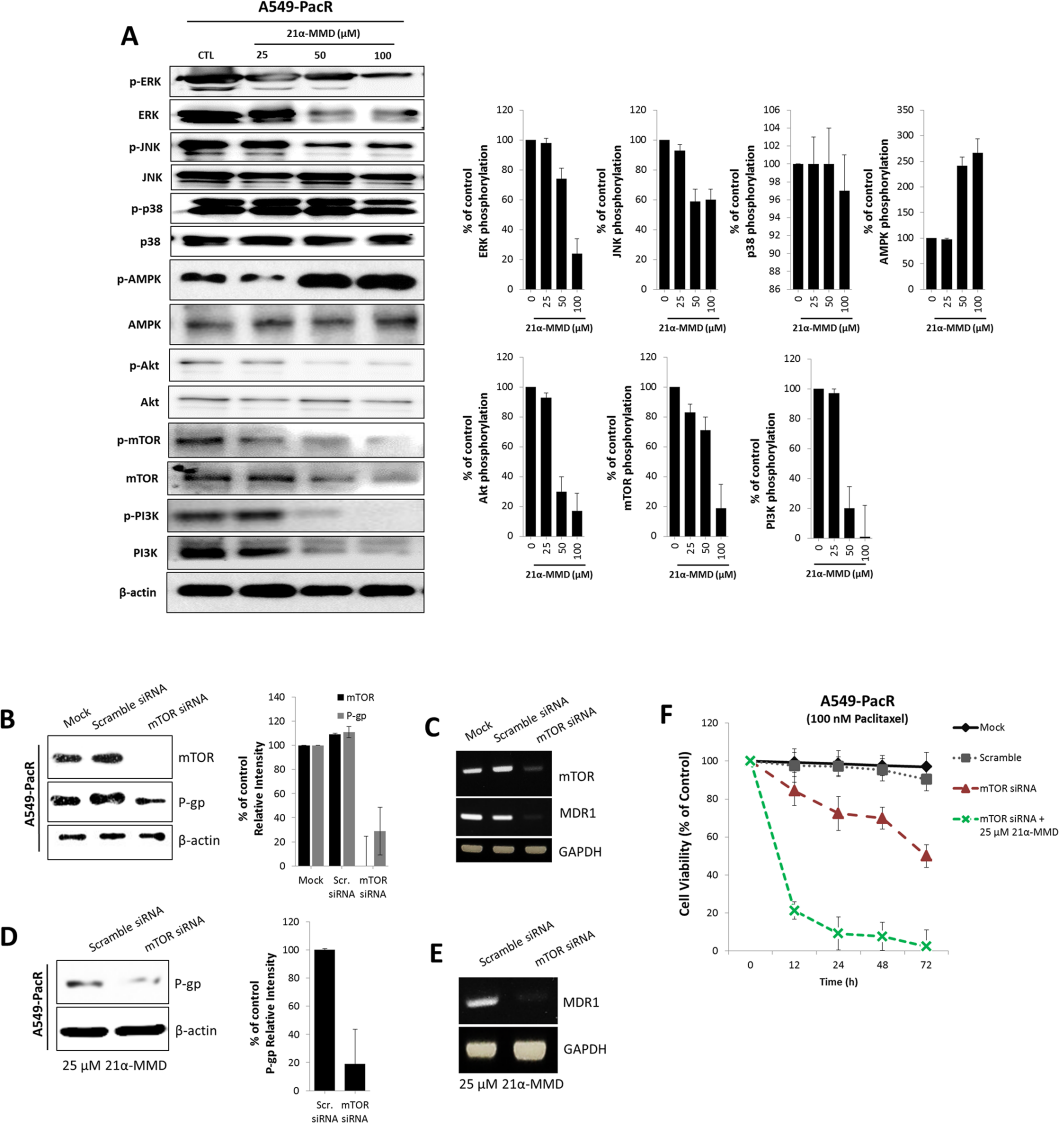
Subsequent downregulation of P-gp/MDR1 expression and inhibitory enhancement of 21α-MMD by stimulating mTOR and related signaling. (A) A549-PacR cells were incubated with 21α-MMD (25–100 μM) for 24 h. Whole-cell lysates were subjected to Western blot analysis using anti-ERK, anti-JNK, anti-p38, anti-Akt, anti-mTOR, anti-PI3K, anti-AMPK and antibodies related to their phosphorylated forms. The β-actin and phospho-protein relevant to the total protein bands confirmed the integrity and equal loading of total protein and phospho-proteins respectively. All protein levels were normalized to the β-actin levels. (B and C) Effect of mTOR knockdown was established first by transiently transfecting mTOR siRNA to A549-PacR cells for 24 h. Scramble siRNA was used as control separated from the mock control. mTOR and MDR1/P-gp protein and mRNA gene expressions were examined by Western blotting and PCR analysis respectively. (D and E) Cells were transiently transfected with mTOR siRNA or Scramble siRNA for 24 h followed by a 24 h exposure to 25 μM 21α-MMD. MDR1/P-gp protein and mRNA gene expression levels were confirmed by Western blotting and PCR analysis respectively. (F) After mTOR siRNA or Scramble siRNA transfections, cells were treated with 100 nM paclitaxel for various time courses and mTOR siRNA was subsequently incorporated with 25 μM 21α-MMD followed by cell viability assessment through MTT assay.

## References

[1] Aldonza MBD, Hong J-Y, Bae SY, Song J, Kim WK, Oh J, et al (2015) Suppression of MAPK Signaling and Reversal of mTOR-Dependent MDR1-Associated Multidrug Resistance by 21α-Methylmelianodiol in Lung Cancer Cells. PLoS ONE 10(6): e0127841 doi:10.1371/journal.pone.0127841 26098947 10.1371/journal.pone.0127841 PMC4476707

