# Supplementary figures and images for: Suppression of MAPK Signaling and Reversal of mTOR-Dependent MDR1-Associated Multidrug Resistance by 21α-Methylmelianodiol in Lung Cancer Cells
Source: PLoS One. 2015 Jun 22;10(6):e0127841. doi: 10.1371/journal.pone.0127841 (PMC4476707; doi:10.1371/journal.pone.0127841)

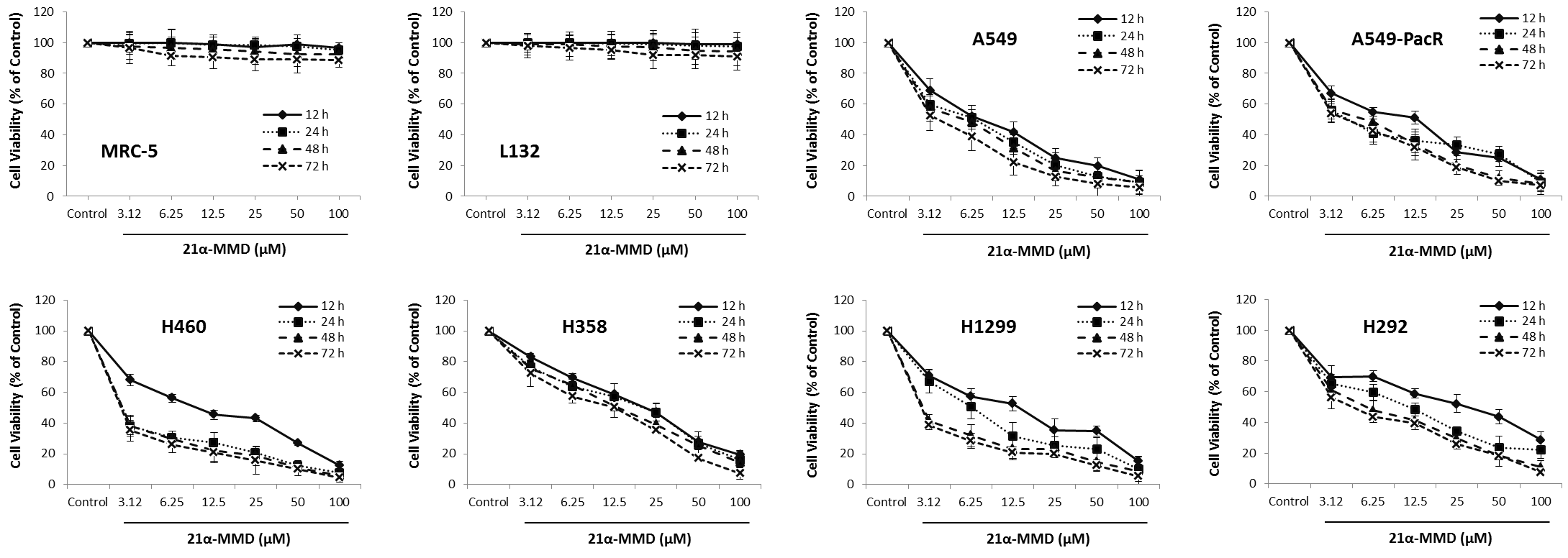

Supplement: S1 Fig — L132 and MRC-5 human normal lung cell lines; A549, A549-PacR, H460, H358, H1299, and H292 human lung cancer cell lines were treated with varying micromolar concentrations of 21α-MMD in a time course analysis as indicated. Cell growth was analyzed by MTT assay and plotted as a percentage. The mean values ± SD (n = 3) are shown. Values are compared to the corresponding control value. (TIF) [file pone.0127841.s001.tif]
